# Use, applicability and reliability of depth of hypnosis monitors in children - a survey among members of the European Society for Paediatric Anaesthesiology

**DOI:** 10.1186/s12871-018-0503-y

**Published:** 2018-04-16

**Authors:** Yuen Man Cheung, Gail Scoones, Robert Jan Stolker, Frank Weber

**Affiliations:** Department of Anaesthesiology, Erasmus University Medical Centre - Sophia Children’s Hospital, Room H-1273, P.O. box 2040, 3000 CA Rotterdam, the Netherlands

**Keywords:** Child, Consciousness monitors, Infant, Surveys and questionnaires

## Abstract

**Background:**

To assess the thoughts of practicing anaesthesiologists about the use of depth of hypnosis monitors in children.

**Methods:**

Members of the European Society for Paediatric Anaesthesiology were invited to participate in an online survey about their thoughts regarding the use, applicability and reliability of hypnosis monitoring in children.

**Results:**

The survey achieved a response rate of 30% (*N* = 168). A total of 138 completed surveys were included for further analysis. Sixty-eight respondents used hypnosis monitoring in children (Users) and 70 did not (Non-users). Sixty-five percent of the Users reported prevention of intra-operative awareness as their main reason to apply hypnosis monitoring. Among the Non-users, the most frequently given reason (43%) not to use hypnosis monitoring in children was the perceived lack or reliability of the devices in children. Hypnosis monitoring is used with a higher frequency during propofol anaesthesia than during inhalation anaesthesia. Hypnosis monitoring is furthermore used more frequently in children > 4 years than in younger children. An ideal hypnosis monitor should be reliable for all age groups and any (combination of) anaesthetic drug. We found no agreement in the interpretation of monitor index values and subsequent anaesthetic interventions following from it.

**Conclusions:**

Prevention of intraoperative awareness appears to be the most important reason to use hypnosis monitoring in children. The perceived lack of reliability of hypnosis monitoring in children is the most important reasons not to use it. No consensus currently exists on how to adjust anaesthesia according to hypnosis monitor index values in children.

**Electronic supplementary material:**

The online version of this article (10.1186/s12871-018-0503-y) contains supplementary material, which is available to authorized users.

## Background

With the introduction of processed electroencephalography, about 20 years ago, the electroencephalogram (EEG) became feasible to be used to easily monitor depth of hypnosis (DoH) in patients receiving general anaesthesia [1]. Whether or not DoH-monitors (DoH-M) have a beneficial impact on peri-operative outcomes, remains subject to discussion [2]. What all currently commercially available DoH-M have in common is that they have been developed for use in adult patients. Clear recommendations regarding the use of the currently available DoH-monitors in paediatric patients are still lacking [3].

The Paediatric Anaesthesia Research Group at Sophia Children’s Hospital in Rotterdam designed and launched an online survey [4] to assess the thoughts of the members of the European Society for Paediatric Anaesthesiology (ESPA) regarding the use, applicability and reliability of DoH-monitoring in children. Besides general aspects regarding the use of DoH-M in children, we were also interested in the thoughts of ESPA members regarding the requirements of an ideal paediatric DoH-M and whether demographic characteristics of the anesthesiologist (age, working experience, etc.) influenced their vision regarding DoH-monitoring in children.

## Methods

According to the Dutch regulations, questionnaire research does not fall under the scope of the Medical Research Involving Human Subjects Act (WMO), as declared by the Central Committee on Research Involving Human Subjects (http://www.ccmo.nl/en/questionnaire-research). Therefore, formal ethics approval was deemed unnecessary according to national regulations and was not obtained.

During the development of the survey, it was evaluated and tested by anaesthesiologists of our paediatric anaesthesia department. The survey consisted of two major parts, beginning with questions concerning the respondents’ demographics, workplace, annual personal case-loads and availability of DoH-M at their institutions. The second part was related to the thoughts of the respondents regarding their personal practice of DoH-monitoring in children and their thoughts about paediatric DoH-monitoring in general. In order to minimize possible bias, the order of the answers to any of our multiple-choice questions were randomized for each respondent. The entire survey is available as supplementary content (see Additional file 1).

On our request, ESPA invited their members (*N*  = 553) by email to participate in our survey. A single reminder was send by e-mail three weeks after the initial invitation. The survey was accessible online in the period from June 28 2013 until August 18 2013.

### Statistical analysis

Respondents were allocated to two groups; “Users” and “Non-users” of DoH-M in children. Non-users were excluded from further analysis when their only reason to not use DoH-M in children was due to the unavailability of a DoH-M in their institution since this was considered a circumstantial reason rather than a personal choice. For nominal data Pearson’s Chi-Square or Fisher’s test were used to analyse the differences between DoH-M Users and Non-users. When needed, data was recoded to maintain a minimum expected count of five to facilitate the Pearson’s Chi-Square or, if applicable, the Fisher’s Exact test. The Mantel-Haenszel test [5], labelled as a “Linear-by-Linear Association” in SPSS, was used for ordinal data (e.g. work experience, age or frequency of giving anaesthesia to certain age groups). *P*-values < 0.05 were considered statistically significant.

The margin of error for our survey data, incl. a 95% confidence level was computed using an online-tool provided by SurveyMonkey [4]. The margin of error is an estimate of the appropriateness of the sample size to represent the whole population (ESPA members).

All analyses were performed using SPSS (IBM SPSS Statistics, version 21).

## Results

We received a total of 168 (30%) responses, of which 14 were incomplete and excluded from analysis. Sixteen respondents didn’t use DoH-M in children due to the unavailability of any DoH-M in their institution and were excluded from further analyses. The margin of error of our sample size was 6%.

Our respondents came from 40 different countries. To present the data in a more comprehensible manner, we categorized them into continents. The majority (*N* = 115; 83%) came from Europe. Baseline characteristics, i.e. professional title, age, type of institution they work in, years of experience in anesthesiology, of the Users (*N* = 68) and Non-users (*N* = 70) are summarized in Table 1.

**Table 1:** Respondents’ baseline characteristics

	Users (*N* = 68)	Non-users (*N* = 70)	*P*-value
Professional title			0.366^a^
Anaesthesiologist	67 (99%)	66 (94%)	
Anaesthesiologist in training (resident)	1 (1%)	4 (6%)	
Practicing in			n/a
Europe	57 (84%)	58 (83%)	
Middle East	6 (9%)	4 (6%)	
East Asia	1 (1%)	2 (3%)	
Australia	1 (1%)	3 (4%)	
South Americas	2 (3%)	1 (1%)	
North Americas	1 (1%)	2 (3%)	
Works in			0.064^a^
(university) children hospital	41 (60%)	31 (44%)	
non-children’s hospital	27 (40%)	39 (56%)	
Years of practice			0.898^b^
< 10 years	17 (25%)	20 (29%)	
11–20 years	27 (40%)	24 (34%)	
> 20 years	24 (35%)	26 (37%)	
Age			0.908^b^
< 40 years	20 (29%)	20 (29%)	
41–50 years	25 (37%)	28 (40%)	
> 51 years	23 (34%)	22 (31%)	

Comparison of baseline characteristics of respondents either using (Users) or not using (Non-users) depth of hypnosis monitoring in children

^a^Fisher’s Exact test

^b^Mantel-Haenszel test

The workplace distribution was 60% children’s hospital and 40% general hospital among DoH-M Users. For the Non-users the distribution was 44% children’s hospital and 56% general hospital. Though not reaching statistical significance (Fisher’s exact test, *p* = 0.064), these results indicate a weak evidence that anaesthesiologists working in children’s hospitals are more likely to use DoH-M than those working in general hospitals.

Both Users (94%) and Non-users (86%) were “most” familiar with the Bispectral Index (BIS) monitor (*p* = 0.09), followed by Entropy (Users 37%, Non-users 26%; *p* = 0.11), the Narcotrend (Users 18%, Non-users 17%; *p* = 0.56) and the AEP-monitor/2 (Users 13%, Non-users 10%; *p* = 0.37). The BIS monitor was used most frequently (77%), followed by Entropy (10%), Narcotrend (6%) and the Cerebral State Index (4%).

In order of descending frequency, DoH-M was used during major surgery (96%), neurosurgery (53%), minor surgery (32%), cardiac surgery (22%) and procedural sedation (19%).

A total of 70 respondents reported to never use DoH-M in children. The majority of them (49%) reported that they think DoH-M was unreliable and/or not validated for use in children. Other reasons were that using a DoH-M wouldn’t affect their method of anaesthesia (30%) and the cost of using DoH-M (24%).

**Fig. 1:**
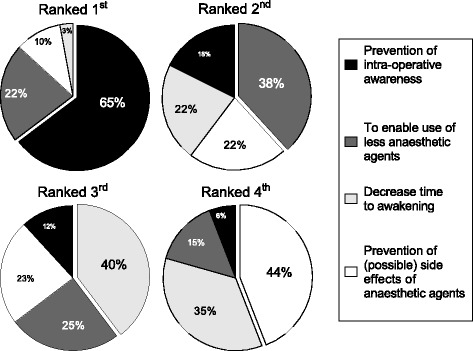
Reasons for hypnosis monitoring. Percentage Users reported their reasons to use depth of hypnosis monitoring in children ranked 1st, 2nd, 3rd and 4th

Prevention of intraoperative awareness was the most frequently reported primary reason to apply DoH-M, whereas preventing (possible) side effects of anaesthetic agents were most frequently reported as least relevant (for details see Fig. 1).

**Fig. 2:**
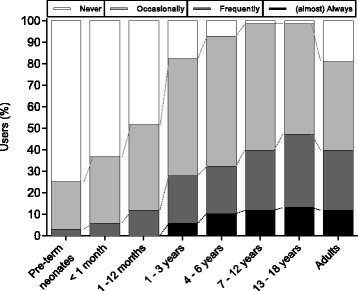
Hypnosis monitoring and age. Patient population in which depth of hypnosis monitoring is being used

The frequency of using DoH-M ranged from 25% in pre-term infants to 98% in teenagers. About 10% of the Users reported to apply DoH-M almost always in patients older than 4 years. Details are given in Fig. 2.

**Fig. 3:**
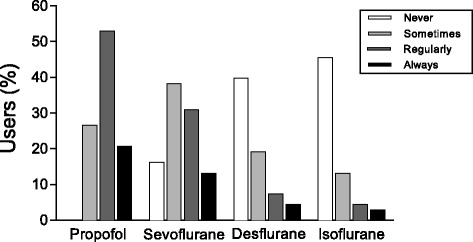
Hypnosis monitoring and anaesthetic. Percentage respondents who “never”, “sometimes”, “regularly” or “always” use depth of hypnosis monitoring with different anaesthetics

All Users reported to use DoH-M during propofol anaesthesia. DoH-M was less frequently used during inhalation anaesthesia (see Fig. 3).

Being asked whether either the actual value of a DoH-Index or its trend over time best reflect the DoH, 62% of the Users preferred to rely on a combination of the actual index value and its trend. Such a combination would result in various drug interventions, such as increasing the hypnotic agent concentration (27%), analgesic agent application (3%), or both (60%), while 10% would not react without additional changes in physiological parameters, i.e. heart rate or blood pressure. Twenty-nine percent of the Users found the DoH best represented by the trend. In the case of an increasing trend they would increase the hypnotic drug concentration (35%), or give additional analgesic drugs (4%) or both (46%), while 13% would only react to the increasing trend when combined with changes in physiological parameters. Another 7% relied only on increases of the actual DoH-index value, resulting in increasing hypnotic drug concentration (24%), additional analgesic drug application (3%) or both (41%), with 31% of them also requiring physiological alterations for an intervention (1% answered “other”).

**Fig. 4:**
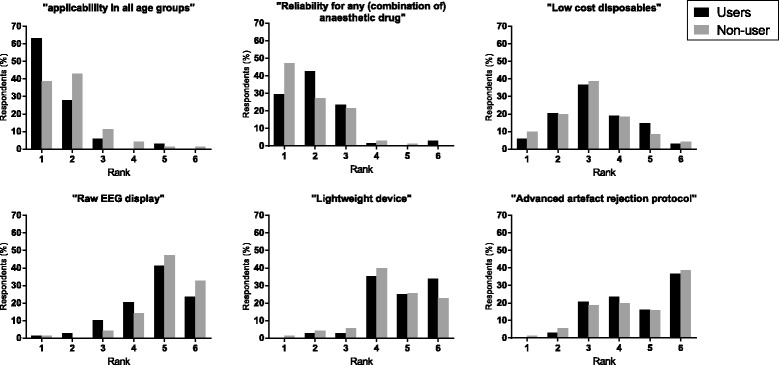
The ideal hypnosis monitor. Features of an ideal depth of hypnosis monitor ranked 1st, 2nd, 3rd, 4th, 5th and 6th by percentage Users and Non-users

According to all respondents, applicability in all patient age groups, reliability for any (combination of) anaesthetic drug, and low-cost disposables were the three most important requirements of a theoretical ideal DoH-M. For more details see Fig. 4.

Eighty percent of the respondents (*N* = 110) agreed that there is a need for a monitor which specifically measures analgesia. Fourteen of the respondents (10%) agreed to the need for a separate analgesia monitor, 43 (31%) preferred a combined analgesia/DoH-M monitor and 53 (38%) agreed to both options. Another fourteen (10%) respondents held a neutral position (“not knowing”) and 14 (10%) disagreed with both types of analgesia monitors. With respect to their thoughts about the need for analgesia monitoring devices, a Mantel-Haenszel test revealed that Users are more optimistic towards it (*p* = 0.04), while no evidence of a difference between DoH-M Users and Non-users regarding their thoughts about a stand-alone analgesia monitor (*p* = 0.63) or a combined DoH/analgesia-monitor (*p* = 0.12) was observed.

## Discussion

Practicing anaesthesiologists dedicated to paediatric anaesthesia perceive the avoidance of intraoperative awareness as the most important reason to use DoH-M in children. The most cited reasons of not using DoH-M in children were serious concerns regarding the reliability of the currently available devices in paediatric patients.

This survey gives an overview of the thoughts and attitudes of (European) anaesthesiologists affiliated with the ESPA concerning the use of DoH-M in children.

Not unexpectedly, the BIS monitor was the device most widely available, regardless of the personal preference to use it or not. Working experience (Table 1) and familiarity with DoH-M were not related to its use in children.

As expected, DoH-M was most often applied in older children, whereas its use in (preterm) neonates was infrequent (see Fig. 2). This pattern is in accordance with a recommendation made by Davidson [3], who reported increasing evidence that DoH-M devices do not work in infants, while there is also increasing evidence they may work in older children.

Interestingly, despite the absence of scientific publications investigating the effect of DoH-M on the incidence of intraoperative awareness in children, this remains the most common indication reported by DoH-M Users to apply this technology. What we currently know, is that the incidence of awareness in children (approximately 1% [6]) is significantly higher than in adults (approximately 0.1–0.2% [7]). In addition, the big trials performed in adult patients investigating the impact of BIS monitoring on the incidence of awareness showed conflicting results, reporting both a reduction of awareness cases [7] and no beneficial effect [8]. Use of less anaesthetics and decreased time to awakening, both reported in paediatric studies [9–12], were ranked 2nd and 3rd in the decision finding process to use DoH-M. At least 44% of the Users chose “prevention of (possible) side effect of anaesthetic agents” as the least important argument for using DoH-M. Bearing in mind the ongoing discussion about the safety and possible neurotoxicity of anaesthetic drugs in the developing brain [13–15], we regard this as an unexpected finding.

Not surprisingly, 39% of the Non-Users chose “Applicability in all age groups” as their most important feature of a hypothetical ideal DoH-M. Users on the other hand chose “prevention of intra-operative awareness” and “To enable use of less anaesthetic agents” as their main reason to use DoH-M in children. These opinions were also reflected by their preferences regarding the most important features of an ideal DoH-M, i.e. “Applicability in all age groups” and “Reliability for any (combination of) anaesthetic drug”.

Index values are helpful and practical to make the EEG understandable during anaesthesia. However, subtle EEG-information will be lost. With no doubt, a raw EEG display on a DoH-M could contribute to assessing the DoH, under the prerequisite that the anaesthesiologist has at least some basic knowledge of clinical encephalography [16]. The latter applies only to a minority of clinical anaesthesiologists. Therefore, it is not at all surprising that this feature was ranked only 5th by most of the respondents.

All Users applied DoH-monitoring, with frequencies varying from “sometimes” to “always” during propofol anaesthesia. This is in accordance with recent UK guidelines published by the National Institute for Health and Care Excellence (NICE), recommending the use of DoH monitoring in all patients receiving total intravenous anaesthesia [17]. DoH-M was used much less frequently during inhalation anaesthesia. This could be due to the fact that it is nowadays well known that end-tidal concentrations of inhalation anaesthetics are closely linked to the likelihood of being awake. For paediatric patients the minimal alveolar concentration of sevoflurane associated with wakefulness (MAC_awake_) has been found to be as low as 0.2–0.3% [18].

The survey also showed disparities in how to interpret the index values and how to intervene. While the device manufacturers typically advise to keep the values of their DoH-Index within a predefined range, the majority of our respondents (62%) believed that the combination of the actual index value and its trend best indicates DoH. In a recent study, performed in adult patients, Schneider et al. [19] demonstrated that combining the BIS with standard anaesthesia parameters (i.e. heart rate) resulted in a prediction probability [20] value of 1.0 to detect consciousness. This suggests that this combination is the perfect indicator of DoH; at least when assuming DoH equals losing and regaining consciousness.

Being asked how to react on increasing DoH-index values, our respondents’ answers showed a huge variability, ranging from increasing the hypnotic drug concentration, giving additional analgesic drugs, increasing both hypnotics and analgesics or even deciding not (yet) to intervene at all. An analgesia monitor could assist in deciding which intervention is probably needed and most respondents agreed with the need for an analgesia monitor.

Since the majority of the ESPA members did not voice their opinions (30% response rate), we have to bear in mind that the results of this survey could be biased. On the other hand, the relatively low margin of error indicates that our sample size represents 95% of the all ESPA members with a +/− 6% margin. The low response rate can be regarded as a result in its own right. This could be interpreted as if the majority of paediatric anaesthesiologists have either significant reservations regarding the reliability and/or applicability of DoH-M in children or, more generally a low level of interest in this subject. We cannot claim to present data which is representative for the European paediatric anaesthesiology community. Nonetheless, we still consider our results relevant, because they very well reflect the tenor of the usual informal inter-collegial conversation regarding paediatric DoH-M during conferences or daily practice.

There is at least a theoretical possibility that respondents who did not have DoH-M available at their institutions would have favoured use of these devices, if given the choice. The design of our survey did not take into account this possibility, which could be regarded as a shortcoming. On the other hand, it would not be correct to assign these respondents to the Non-user group, which consisted by default of respondents who had DoH-M available but decided not to use them in children.

As long time users of various DoH-monitoring devices in children we would like to share our vision on this controversial topic with our readers and provide the following recommendations: In accordance with the current UK NICE guidelines [17] we highly recommend the use of DoH-monitoring during propofol anaesthesia in all paediatric patients beyond infant age [3]. In children receiving inhalational anaesthesia we recommend the use of DoH-monitoring devices which provide the anaesthesiologist with additional information regarding the raw-EEG. This information is vital to prevent the child, in particular of the youngest age group, from EEG burst suppression patterns, indicating anaesthetic drug overdose.

Future research in this field should focus on the youngest patient age group. A very promising recent approach is the interpretation of the EEG power spectrum, displayed as Density Spectral Array (DSA). The major advantage of DSA is that it uses raw-EEG information in real time and that drug specific EEG-signatures have been identified [21], even for paediatric patients [16, 22]. This new technology is already implemented in several commercially available DoH-monitors.

## Conclusions

In conclusion, for ESPA affiliated anaesthesiologists who filled in our survey, prevention of intraoperative awareness was the most important reason to use DoH-M in children. The perceived lack of reliability of the currently available devices, when used in children, was the most important reason for not using DoH-M. No consensus currently exists on how to adjust anaesthesia according to DoH-M indices in children. According to the respondents to this survey an ideal DoH-M should be reliable for all age groups and any (combination of) anaesthetic agent.

## Additional file

Additional file 1: The survey as presented to our respondents. (PDF 341 kb)

## Abbreviations

BIS: Bispectral Index
DoH: Depth of hypnosis
DoH-M: Depth of hypnosis monitors
DSA: Density Spectral Array
EEG: Electroencephalogram
ESPA: European Society for Paediatric Anaesthesiology
MAC_awake_: Minimal alveolar concentration associated with wakefulness
NICE: National Institute for Health and Care Excellence
