# Supplementary material for: Use, applicability and reliability of depth of hypnosis monitors in children - a survey among members of the European Society for Paediatric Anaesthesiology
Source: BMC Anesthesiol. 2018 Apr 16;18:40. doi: 10.1186/s12871-018-0503-y (PMC5902980; doi:10.1186/s12871-018-0503-y)
Supplement: Supplementary file 1 — The survey as presented to our respondents. (PDF 341 kb) [file 12871_2018_503_MOESM1_ESM.pdf]

Dear colleague,

Thank you very much for agreeing to complete our survey on depth of anaesthesia monitoring in children.
Your input is highly appreciated.
We estimate that it will take you approximately 7 minutes to complete the survey.

Sincerely,

Yuen M. Cheung
Frank Weber

Paediatric Anaesthesia Unit
Sophia Children's Hospital
Erasmus University Medical Center
Rotterdam
The Netherlands

e-mail: paediatric.anaesthesia.research@erasmusmc.nl

1. What is your professional title

- Anaesthesiologist
- Anaesthesiologist in training (resident)
- Nurse Anaesthetist
- Physician Assistant
- Other (please specify)

2. What is your age?

- <30 years
- 30-40 years
- 41-50 years
- 51-60 years
- >60 years

3. In which country are you presently working?

4. In which hospital do you give your most anaesthetics?

5. How many years have you been practicing anaesthesiology?

	<5	5-10	11-20	>20
Years of practice	○	○	○	○

6. How often do you give anaesthesia for the following different patient age groups?

	Never	Occasionally	Frequently
Pre-term neonates	○	○	○
Full-term neonates to 1 month	○	○	○
Infants 1 month to 1 year	○	○	○
1-3 years	○	○	○
4-6 years	○	○	○
7-12 years	○	○	○
13-18 years	○	○	○
Adult patients (> 18 years)	○	○	○

7. How often do you give anaesthesia for the following types of surgery in paediatric patients

	Never	Occasionally	Frequently	(Almost) always
Minor surgery	○	○	○	○
Major surgery	○	○	○	○
Neurosurgery	○	○	○	○
Cardiac surgery	○	○	○	○

8. Which of the following depth of anaesthesia monitors are you familiar with? *(you can choose multiple answers)*

- ☐ Bispectral Index
- ☐ Entropy (Datex Ohmeda/ GE)
- ☐ aepEX
- ☐ cAAI
- ☐ AEP-monitor/ 2
- ☐ Cerebral State Index
- ☐ Narcotrend
- ☐ I don't know any depth of anaesthesia monitor
- ☐ Other (please specify)

[ ]

9. Which of the following depth of anaesthesia monitors are available at your institution? *(you can choose multiple answers)*

- ☐ Bispectral Index
- ☐ Entropy (Datex Ohmeda/ GE)
- ☐ aepEX
- ☐ cAAI
- ☐ AEP-monitor/ 2
- ☐ Cerebral State Index
- ☐ Narcotrend
- ☐ There are no depth of anaesthesia monitors in my institution
- ☐ Other (please specify)

10. Do you use depth of anaesthesia monitoring in pediatric patients?

- ○ Yes
- ○ No

11. What is/are your reason(s) for not using depth of anaesthesia monitoring in paediatric patients? *(you can choose multiple answers)*

- ☐ It's too expensive
- ☐ It's unreliable
- ☐ It doesn't effect my method of anaesthesia
- ☐ No particular reason
- ☐ Other (please specify)

12. How often do you use depth of anaesthesia monitoring in the following age groups?

	Never	Occasionally	Frequently	(Almost) always
Pre-term neonates	○	○	○	○
Full-term neonates to 1 month	○	○	○	○
Infants > 1 month < 1 year	○	○	○	○
1-3 years	○	○	○	○
4-6 years	○	○	○	○
7-12 years	○	○	○	○
13-18 years	○	○	○	○
Adult patients (> 18 years)	○	○	○	○

13. For which of the following procedures do you use depth of anaesthesia monitoring? *(you can choose multiple answers)*

- ☐ Minor surgery
- ☐ Procedural sedation
- ☐ Major surgery
- ☐ Cardiac surgery
- ☐ Neurosurgery
- ☐ Other (please specify) ________

14. Please rank the following monitors in order from those most to least frequently used in your personal practice *(you can drag and drop the options)*

[▾]	Bispectral Index	☐ Not available
[▾]	Entropy (Datex Ohmeda/ GE)	☐ Not available
[▾]	aepEX	☐ Not available
[▾]	cAAI	☐ Not available
[▾]	AEP-monitor/ 2	☐ Not available
[▾]	Cerebral State Index	☐ Not available
[▾]	Narcotrend	☐ Not available

15. Please rank the following reasons for using depth of anaesthesia monitoring in order from the most to least important for you ***(you can drag and drop the options)***

- To enable use of less anaesthetic agents
- Prevention of intra-operative awareness
- Decrease time to awakening
- Prevention of (possible) side effects of anaesthetic agents

16. Do you have any other reasons for using depth of anaesthesia monitoring?

- Yes
- No

17. What is/are your additional reason(s), in order of decreasing importance, for using depth of anaesthesia monitoring in paediatric patients?

Reason 1

Reason 2

Reason 3

Reason 4

18. Which aspect of the index value do you think best indicates to the depth of anaesthesia?

- The trend (i.e. decreasing trend or increasing trend)
- The exact values
- The trend and exact values (depends on the monitor)
- Other (please specify)

19. How would you intervene if ONLY the trend of the index values is increasing?

- Increase the hypnotics
- Increase analgesics
- Combination of hypnosis and analgesics
- Do nothing; I only intervene when also other variables change (e.g. resp rate, pulse, bp etc)
- Other (please specify)

20. How would you intervene if ONLY the exact value of the index values is too high?

- Increase the hypnotics
- Increase analgesics
- Combination of hypnosis and analgesics
- Do nothing; I only intervene when also other variables change (e.g. resp rate, pulse, bp etc)
- Other (please specify)

21. How would you intervene if the index value is increasing and too high?

- Increase the hypnotics
- Increase analgesics
- Combination of hypnosis and analgesics
- Do nothing; I only intervene when also other variables change (e.g. resp rate, pulse, bp etc)
- Other (please specify)

22. With which of the following anaesthetic drugs do you use depth of anaesthesia monitoring in paediatric patients?

	Never	Sometimes	Regularly	Always	Not applicable
propofol					
sevoflurane					
desflurane					
isoflurane					
halothane					

23. Do we need the following devices in paediatric anaesthesia?

	Completely disagree	Disagree	I don't know	Agree	Completely agree
Separate analgesia monitor					
Combined analgesia & depth of hypnosis monitor					

24. Please rank the following requirements for your ideal depth of anaesthesia monitor in the order from those most (1) to least (6) important. ***(you can drag and drop the options)***

- [ ] Applicability in all age groups
- [ ] Low costs disposables
- [ ] Reliability for any (combination of) anesthetic drug
- [ ] Lightweight device
- [ ] Raw EEG display
- [ ] Advanced artefact rejection protocol

This was our last question. We thank you for taking the time to fill in our survey.
